# Split-Thickness Skin Grafting for the Management of Traumatic Pretibial Hematomas

**DOI:** 10.7759/cureus.72340

**Published:** 2024-10-24

**Authors:** Boris Joutovsky, Patrizio Petrone, Daphnee Beaulieu, Jerry Rubano, Gerard A Baltazar

**Affiliations:** 1 Department of Surgery, NYU Grossman Long Island School of Medicine, Mineola, USA; 2 Department of Surgery, NYU Langone Hospital - Long Island, Mineola, USA; 3 Department of Surgery, Long Island Community Hospital, Patchogue, USA

**Keywords:** pretibial lesion, skin grafting, subcutaneous hematoma, tension hematoma, traumatic pretibial hematoma

## Abstract

Pretibial traumatic hematomas, a subtype of subcutaneous tension hematomas, are a frequent but understudied injury seen predominantly among the elderly. This patient cohort has a high incidence of comorbidities and frailty. They are frequently taking antiplatelet medications and systemic anticoagulants. The treatment of these injuries can be costly and associated with significant morbidity and even mortality. Early detection and treatment are important when managing pretibial hematomas with the potential for skin necrosis. We report on a case where we performed how early operative debridement, negative pressure wound therapy, and subsequent split-thickness tissue grafting may be an effective management strategy for pretibial hematomas and suggest the importance of establishing standardized institutional approaches for their management.

## Introduction

Subcutaneous tension hematoma is a traumatic, and rarely spontaneous, condition frequently seen in the geriatric population. Patients commonly present to the emergency department with multiple comorbidities and are often on anticoagulation medications [1-3]. The injury and subsequent disease process contribute to significant hospital costs and prolonged hospital stays and often result in delayed operative care [4].

Traumatic mechanisms, including blunt or shearing forces, disrupt the underlying skin vasculature and cause blood to accumulate underneath the skin. This pressure can exceed capillary perfusion pressure and result in skin ischemia with subsequent necrosis [1]. Skin conditions such as dermatoporosis are seen in the aging population and result in skin atrophy, fragility, and decreased elasticity, predisposing the geriatric population to an increased risk of hematoma formation and subsequent tissue necrosis [5].

Releasing the underlying tension is the main goal when managing subcutaneous tension hematomas, with aspiration previously described as a potential initial step [6]. The development of underlying blood clots may make aspiration difficult; thus, incision and drainage are preferred, especially if the skin has become ischemic or necrotic. If hemodynamically stable, the patients can be managed with operative incision and drainage and excisional debridement. Split-thickness skin grafting (STSG), either at the time of debridement or several days following debridement to allow for improvement of wound bed quality, is an important management strategy that allows for complete wound healing of the subcutaneous tension hematoma [7].

Despite the high incidence of this traumatic condition, there exists scarce literature comparing the different therapeutic approaches for managing subcutaneous tension hematomas. Traumatic pretibial hematomas are a subtype noted frequently at our institution but for which published literature provides little guidance for optimal management. For example, traumatic pretibial hematoma is seldom described as an independent pathology and is often characterized in association with pretibial lacerations. The terminology that describes the condition in these studies is not standardized with pretibial hematomas described as deep dissecting, superficial, expanding spontaneous, tension subcutaneous, or closed leg hematomas.

The traumatic pretibial hematoma, similar to other subcutaneous tension hematomas, may result in skin necrosis and potential infectious complications, progressive tissue loss, and extended lengths of stay [4]. Particularly among the elderly population, in order to minimize tissue loss, morbidity, mortality, cost, and length of stay, an evidence-based approach to managing this condition should be developed [8]. We describe in detail an archetypal case of traumatic pretibial hematoma successfully treated at our institution with operative debridement, negative pressure wound therapy, and staged split-thickness skin graft. We review some of the existing evidence and advocate for the development of a standardized institutional management strategy. We will present our established practice guidelines.

## Case presentation

**Figure 1:**
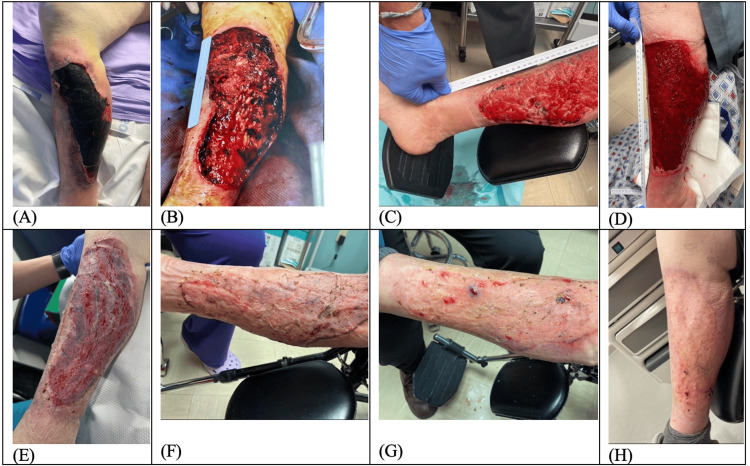
Left lower extremity pretibial subcutaneous tension hematoma treatment progression. A. Patient presentation in the emergency department, demonstrating skin necrosis overlying traumatic hematoma. B. Intraoperative debridement of skin, subcutaneous, and fascial layers. C. Outpatient post-operative follow-up after xenograft and negative pressure wound vacuum therapy. D. One-month post-injury, healthy granulation layer. E. Post-operative office visit following split-thickness skin grafting. F. Ten-day follow-up demonstrating near 100% take of skin graft.  G. Six weeks post skin graft. H. Four months since skin graft.

An 80-year-old female, with a history of recurrent falls, presented from the nursing facility for right leg traumatic hematoma and skin necrosis. She has an additional past medical history of atrial fibrillation (CHAD-VASc score 7) and lower extremity deep vein thrombosis (DVTs) on Eliquis anticoagulation. Physical exam upon presentation was consistent with right lower extremity (RLE) hematoma approximately 25cm x 15cm x 2cm with sloughing and skin breakdown around the edges of the wounds and overlying complete skin necrosis (Figure 1A). She underwent operative sharp excisional debridement of the skin, subcutaneous fat, and fascia; evacuation of hematoma; and placement of negative pressure therapy wound vacuum (Figure 1B). Three days following index debridement, the patient returned to the operating room for placement of Acell © xenograft (8 grams particulate xenograft, 10cm x 15cm 6 layer xenograft, covered by non-adhesive (Adaptic©, 3M, Maplewood, MN) gauze and water-soluble lubricant) with replaced wound vacuum negative pressure therapy. After the development of granulation tissue at the wound bed at one-week and one-month appointments (Figures 1C-1D), the patient underwent the third and final operative intervention with STSG with the right thigh donor site (Figure 1E). The patient received perioperative cephalosporin antibiotics during her hospital course without complication. The patient had excellent skin healing at outpatient follow-up at 10 days, six weeks, and four months after grafting (Figures 1F-1H). 

## Discussion

Understanding the importance of pretibial hematomas and identifying the best approach to managing them is necessary for decreasing patient mortality, complications, and hospital length of stay among a growing population of elderly patients with chronic anticoagulation [4,7].

Our study highlights the effective management of immediate operative debridement of a subcutaneous tension hematoma with skin necrosis seen in a geriatric patient. Immediate operative debridement allowed for relief of underlying pressure that led to tissue necrosis and for the adequate debridement of the tissue eschar to allow maximal healing potential and minimize the risk of soft tissue infection. The patient’s progression to tissue necrosis prior to arrival to ED combined with her use of anticoagulation had caused the wound to be large in size (25cm x 15cm x 2cm). We therefore used negative pressure therapy to allow for granulation of the wound bed and performed a staged procedure with STSG. Given the presence of extensive necrotic tissue at the time of original debridement, the decision was made to delay grafting until a healthy wound bed was present. Following the STSG, the patient was discharged to a sub-acute rehabilitation facility where she progressed to ambulation with an assistive device. She was seen on multiple occasions in the office and demonstrated excellent wound healing and good functionality of their lower limb.

Patients who suffer from pretibial hematomas are often comorbid and on chronic therapeutic anticoagulation. When early diagnosis is not accomplished, there is often a delay in treatment and development of skin necrosis. Some reports have demonstrated that early drainage in ED allows for optimization if skin grafting is required [6]. Early stages of traumatic pretibial hematomas present with local symptoms of erythema, swelling, and hot skin, which can be confused with cellulitis and erysipelas. This can lead to unnecessary treatments with antibiotic therapy and delayed surgical care [1]. In some cases, treatment may include initial anticoagulation reversal only [2], and often, these lesions are only diagnosed when skin necrosis is present [1]. Although the literature does not indicate an absolute need for antibiotics, we treated our patient with perioperative cephalosporin to reduce the risk of infection from skin flora.

Pretibial hematomas should be among the differential of providers who are treating patients with age-related dermatoporosis. As the epidermis atrophies with age and the vasoelastic properties of subcutaneous vessels diminish, minor traumatic events may predispose patients to hemorrhage within the subcutaneous tissue or deeper within the muscle fascia. Early hematoma evacuation may lead to smaller skin incisions and allow for primary closure [1].

The vast majority of studies looking at the management of pretibial hematomas focus on case reports. A single retrospective study by Salmeron-Gonzalez et al. [4] described 180 patients, compared management outcomes, and clearly outlined their clinical results. They showed that early drainage reduced complication rates and hospital stays. Debridement and second-intention healing in cases with small areas of skin necrosis could prevent hospitalization and further operations. Patients who require coverage surgery may benefit from the procedure performed on the same day as debridement, rather than a two-staged approach - this process may reduce hospitalization time and the incidence of medical complications.

The patient treated at our institution underwent multiple operative debridements and placement of a xenograft for the very large wound. This multi-stage process allowed for excellent STSG take and may be superior for healing larger pretibial hematoma-related wounds - staged formation of an adequate granulation tissue layer contributed to successful STSG in a patient with baseline weak integument.

Seppälä et al. [8,9] studied pretibial hematomas at a large single-center hospital setting over a five-year period. They report that patients with hematomas that required evacuation had higher compromised independence and were more likely to be female, elderly, comorbid, on anticoagulants, and with dermatoporosis. In cases where skin necrosis occurred, wound dressings or negative pressure wound therapy were applied to aid in the formation of granulation tissue for secondary skin defect coverage. Long-term wound care, repeat hospitalization, and repetitive surgery were often needed. Repeat hospitalizations and subsequent need for revision surgery and later skin grafting increased hospital costs.

Early diagnosis and prompt hematoma evacuation are therefore paramount in cutting costs by lessening the need for revision surgery, skin grafting, and hospitalization [9]. Wound care itself provides for increased cost to patients [9]. Other studies have demonstrated that, when compared to hip fractures, pretibial hematomas are associated with a higher percentage of repeat surgery, longer average hospital lengths of stay, and a higher rate of hospital mortality [3]. Prolonged hospitalization may be caused by prolonged wound management, occupational and physical therapy input, social input, or pre-existing comorbidities that require further in-patient medical care [2].

Glass et al. [7] described that the majority of evidence-based studies that guide clinical practice for pretibial lacerations are derived from small retrospective case series, revealing the need for robust, adequately controlled, prospective trials. This study brings up important questions of whether traumatic limb injuries can be evaluated in a standardized manner in order to be generalized across populations. This may also permit comparative data and may allow institutions to establish a standardized treatment plan for traumatic pretibial hematomas. This can educate providers about the necessity for early intervention with the goals of decreasing hospital stay, hospital cost, and the extent of tibial disease to optimize patient recovery. Based on this and other similar cases, our institution is developing a standardized guideline for the management of pretibial hematomas that closely mirrors the progress of this case report.

Preventive measures should also be in place for the geriatric population that will decrease the prevalence of pretibial hematomas. Skin protective clothing, tibial protectors that can sometimes be incorporated into stockings, and a safe home environment that limits sharp corners and blunt furniture can be introduced to the geriatric population. Public transportation officials should be informed about this health problem to provide similar safe travel measures for the elderly [1].

## Conclusions

Traumatic pretibial hematomas are a commonly seen pathology among elderly patients who often have comorbidities and take blood-thinning medications. Rapidly relieving pressure caused by the hematoma and restoring skin coverage and limb functionality remain the mainstays of treatment. Multiple treatment pathways exist, and we recommend operative debridement, and negative pressure wound therapy, including the use of xenograft to accelerate granulation tissue in-growth skin grafting and STSG as a rapid, reasonable, and effective method of managing subcutaneous tension hematomas and suggest that institutions create evidence-based guidelines to expedite care of these patients and maximize positive outcomes.
